# Temperature assistance of electric field-controlled spin–orbit torque-based magnetization switching in PMN–PT/FePt heterostructures†

**DOI:** 10.1039/d1ra00919b

**Published:** 2021-03-24

**Authors:** Qi Guo, Zhicheng Wang

**Affiliations:** School of Materials Science and Engineering, Taiyuan University of Science and Technology Taiyuan 030024 China zcwang@tyust.edu.cn

## Abstract

We report the temperature assistance of electric field (E-field)-controlled spin–orbit torque (SOT)-based magnetization switching of L1_0_-FePt films grown on a PbMg_1/3_Nb_2/3_O_3_–PbTiO_3_ (PMN–PT) (011) substrate, which generates considerable strain *via* piezoelectric effects of the PMN–PT substrate under E-field. Owing to large strain-induced effective field and weak perpendicular magnetic anisotropy (PMA) at a high temperature, E-field controls the PMA- and SOT-based magnetization switching more effectively. Driven by E-field, magnetization switching is detected by a magnetic optical Kerr (MOKE) microscope under a fixed perpendicular magnetic field. Furthermore, E-field modulates change of anomalous Hall resistance regularly, which enables us to achieve the bidirectional transmission of data by designing an E-field controlled SOT-based logical circuit. This study indicates an efficient way to fabricate potential E-field-controlled spintronic applications at high temperatures.

## Abstract 2

E-field has an improved regulating effect on PMA and SOT-based current induced magnetization switching of PMN–PT/FePt heterostructures.

## Graphical abstract

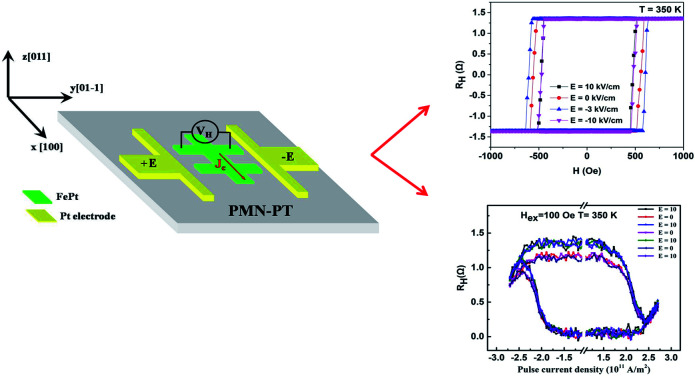

## Introduction

Spin–orbit torque (SOT)-based current-induced magnetization switching has drawn considerable interest due to its prospective utility in low energy consuming spintronic devices.^1–3^ SOT is in a dominant position in research field of spintronics for magnetization switching, which makes it an aplenty and energy efficient physical phenomenon.^4^ The technical significance of SOT for the promotion of future magnetic random access memories has been introduced, which records information *via* SOT-based magnetization switching.^5^ FePt with L1_0_-ordered is an ideal magnetic alloy for information recording with high perpendicular magnetic anisotropy (PMA), the source of which comes from hybridization between Fe 3d and Pt 5d electrons and spin–orbit coupling (SOC). SOC is a precondition of SOT effects.^6–8^

Electric field (E-field)-controlled magnetic and electric properties, which is known as the magnetoelectric (ME) coupling, also have been investigated in ferromagnetic/ferroelectric (FM/FE) heterostructures owing to the advantages of reduced energy consuming, high efficiency and high storage density.^9,10^ Strains generated *via* the piezoelectric effects of a ferroelectric substrate under E-field-controlled magnetic and electric properties is one of the most extensively used methods of ME coupling.^11,12^ The PbMg_1/3_Nb_2/3_O_3_–PbTiO_3_ (PMN–PT) substrate attracted considerable attention due to its high piezoelectric activity in FM/FE heterostructures, which is an ideal strain source.^13^ The PMN–PT(011) substrate has largest ferroelastic domain switching and piezoelectric coefficients *d*_33_ along the [01−1] direction,^14^ which means E-field along the [01−1] direction controls ME coupling in FM/PMN–PT(011) heterostructures more efficiently. E-field-controlled SOT switching and SOT switching logic operations on the PMN–PT substrate also have been demonstrated.^15,16^ In addition, few researches on temperature assistance of SOT-based magnetization switching are reported.^17,18^ Thus, it is rational to explore the mechanism of temperature assistance of E-field controlled SOT-based magnetization switching in PMN–PT/FePt heterostructures.

In this study, we explore the temperature assistance of E-field-controlled SOT-based magnetization switching. E-field has an improved manipulating effect on PMA- and SOT-based magnetization switching of PMN–PT/FePt heterostructures at 350 K. E-field controlled magnetization switching is detected by a magnetic optical Kerr (MOKE) microscope. Then, an E-field controlled SOT-based logical circuit is designed, which is based on the reversible change of anomalous Hall resistance regulated by E-field and realizes the bidirectional transmission of data.

## Experimental method

**Fig. 1:**
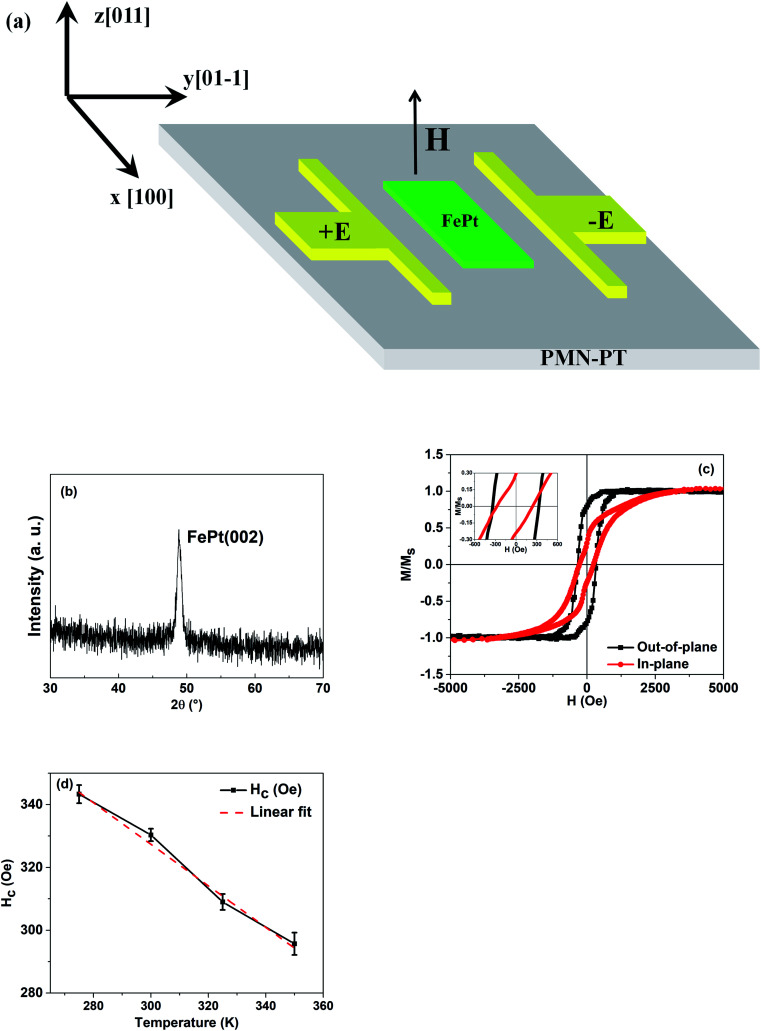
(a) Schematic of the Hall bar used in this study. (b) The GIXRD pattern of FePt films deposited on the PMN–PT substrate. (c) In-plane and out-of-plane *M*–*H* loops of the PMN–PT/FePt heterostructure. The inset shows the enlarged *M*–*H* loops. (d) Out-of-plane coercivity as a function of varying temperatures.

2.5 nm-FePt films were grown on the PMN–PT(011) substrate by magnetron sputtering at 400 °C with a base pressure less than 2 × 10^−6^ Pa. Continuously, FePt films were fabricated into Hall bar *via* standard lithography and Ar ion milling, the size of which is 20 × 120 μm, as shown in Fig. 1(a). The Pt electrodes are used to apply uniform E-field. Before each measurement, PMN–PT substrates were polarized by E-field of 15 kV cm^−1^.

The surface roughness of the PMN–PT(011) substrates was measured by an atomic force microscope (AFM) and the magnetic properties of FePt films were obtained on a vibrating sample magnetometer (VSM). The crystal structure of the FePt films was analyzed *via* grazing incidence X-ray diffraction (GIXRD). Magnetization switching is detected by the MOKE microscope. *R*_H_–*H* and *R*_H_–*I* loops were measured on a physical property measurement system (PPMS) with Keithley 6221, 2400 and 2182A.

## Results and discussion

The root mean square roughness value of the PMN–PT(011) substrate was measured by AFM, which is 0.51 nm (Fig. S1, ESI†). High quality surface of the PMN–PT(011) substrate guarantees the strain transfer efficiency from PMN–PT(011) to FePt films. The GIXRD pattern of the FePt films is shown in Fig. 1(b). The (002) peak of FePt can be observed at about 48.9°, which indicates the L1_0_ phase and the [001] orientation of the FePt films. Fig. 1(c) demonstrates the *M*–*H* loops of FePt films at 275 K and Fig. 1(d) displays the out-of-plane coercivity as a function of varying temperatures for FePt films. The coercivity of FePt films has an obvious change, which decreases from 343 Oe to 295 Oe with the increase in temperature. The reduction of coercivity results from both the increase in thermal fluctuation and decrease in magnetic anisotropy energy with an increase in temperature.^19^ We can draw a conclusion that the L1_0_-FePt films are obtained.

**Fig. 2:**
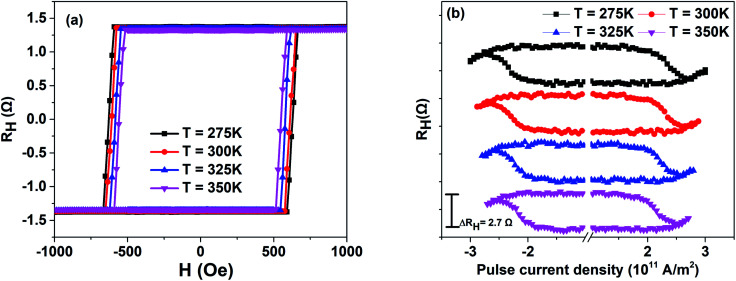
(a) *R*_H_–*H* loops of L1_0_-FePt films under different temperatures. (b) *R*_H_–*I* loops of L1_0_-FePt films with *H*_x_ = 500 Oe under different temperatures.

For the sake of understanding the temperature assistance of PMA and SOT-based magnetization switching, the *R*_H_–*H* loops of L1_0_-FePt films under different temperatures were measured, as shown in Fig. 2(a). Rectangular *R*_H_–*H* loops reconfirm FePt films have strong PMA in the whole temperature range. The coercivity *H*_c_ of *R*_H_–*H* loops decreases monotonically while temperature increases from 275 K to 350 K, which is the same as the coercivity change of Fig. 1(c) and indicates weak PMA at high temperatures. It is noteworthy to notice that there is a slight reduction in the change of the anomalous Hall resistance Δ*R*_H_ with an increase in temperature (Fig. S2, ESI†), Δ*R*_H_ is the out-of-plane magnetization of L1_0_-FePt films,^20^ which decreases with the increase in temperature due to the increasing of thermal fluctuation and decreasing of magnetic anisotropy energy.^19^ Therefore, the corresponding Δ*R*_H_ decreases with the increase in temperature. However, the temperature varies in a small range from 275 K to 350 K, which results in a slight reduction of Δ*R*_H_. For *R*_H_–*I* measurements, the external magnetic field *H*_x_ of 500 Oe and pulse current are applied along the [100] direction. The external magnetic field *H*_x_ is applied to overcome the Dzyaloshinskii–Moriya interaction (DMI) effective field *H*_DMI_, which leads to current-induced magnetization switching.^21,22^ The *R*_H_–*I* loops of L1_0_-FePt films under different temperatures are presented in Fig. 2(b). While the temperature increases, the critical current density *J*_c_ reduces from 2.35 × 10^11^ A m^−2^ to 2.12 × 10^11^ A m^−2^, which can be explained by formula (1):^21^1
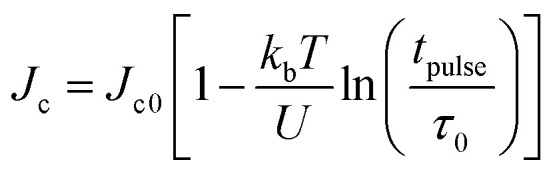
where *J*_c_ is the critical current density, *J*_c0_ is the zero thermal critical current density, *U* is the energy barrier between two magnetization states, *k*_b_ is the Boltzmann constant, *T* is the temperature, *t*_pulse_ is the pulse width, *τ*_0_ = 1 ns. It is clear that the critical current density is inversely proportional to temperature.

**Fig. 3:**
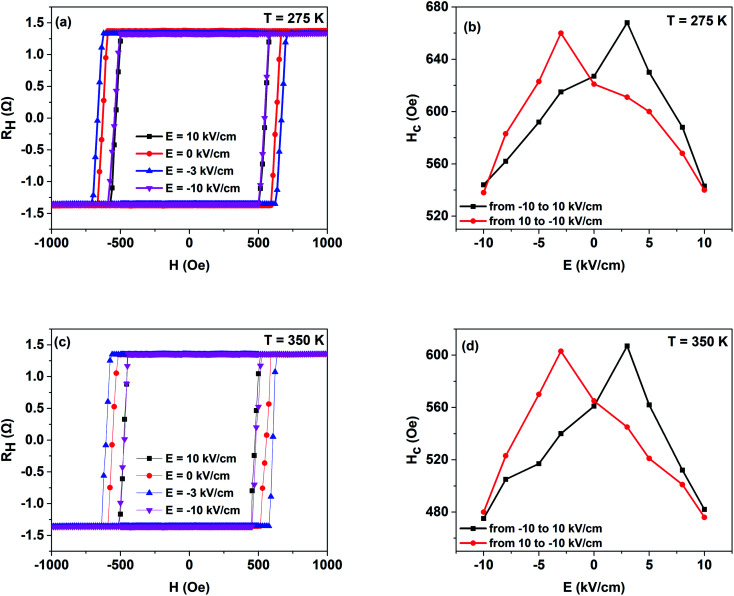
(a) *R*_H_–*H* loops under varying E-fields at 275 K. (b) Dependence of coercivity *H*_c_ on the applied E-field at 275 K. (c) *R*_H_–*H* loops under varying E-fields at 350 K. (d) Dependence of coercivity *H*_c_ on the applied E-field at 350 K.

To survey the temperature assistance of the E-field controlled PMA of PMN–PT/FePt heterostructures, *R*_H_–*H* loops under varying E-field at 275 K were obtained, as shown in Fig. 3(a). E-field generates an apparent change in coercivity and Δ*R*_H_ keeps still. Dependence of coercivity *H*_c_ on the applied E-field at 275 K is summarized in Fig. 3(b). Coercivity *H*_c_ varies along a butterfly shape loop, which presents a coercivity *H*_c_ change of 22.2% from 540 Oe (*E* = 10 kV cm^−1^) to 660 Oe (*E* = −3 kV cm^−1^) and indicates E-field controls PMA of PMN–PT/FePt heterostructures reversibility at 275 K. PMA of L1_0_-FePt originates from both hybridization between Fe 3d and Pt 5d electrons and SOC.^23^ According to the piezoelectric properties under E-field reported by Peng and Li,^13,24^ when the E-field decreases from 10 kV cm^−1^ to −10 kV cm^−1^, L1_0_-FePt films suffer compressive strain along the [01−1] direction at *E* = −3 kV cm^−1^. Consequently, L1_0_-FePt films suffer compressive strain along the [011] direction and tensile strain along the [100] at *E* = −3 kV cm^−1^. The compressive strain along the [011] direction not only improves the chemical ordering parameter S of L1_0_-FePt films, which results in stronger SOC, but also increases the hybridization between Fe 3d and Pt 5d.^7,23,25,26^ As a result, PMA and coercivity *H*_c_ are enhanced at *E* = −3 kV cm^−1^. Similarly, L1_0_-FePt films suffer tensile strain at *E* = ±10 kV cm^−1^ along the [011] direction, which reduces the PMA and coercivity *H*_c_. Then, Fig. 3(c) and (d) show the *R*_H_–*H* loops under varying E-field at 350 K and the dependence of coercivity *H*_c_ on applied E-field at 350 K, respectively. The coercivity *H*_c_ at 350 K also varies along a butterfly shape loop. However, compared with the change in coercivity *H*_c_ at 275 K, the coercivity *H*_c_ has a larger change of 32.3% from 476 Oe (*E* = 10 kV cm^−1^) to 603 Oe (*E* = −3 kV cm^−1^) at 350 K. Higher temperature enhances the piezoelectric coefficient of the PMN–PT(011) substrate and results in a large strain-induced effective field *H*_s_ under E-field, which is given by formula (2):^27^2
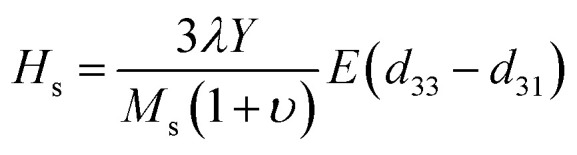
where *M*_s_ is the saturated magnetization (1000 emu cm^−3^), *Y* is the Young's modulus (180 GPa), *υ* is the Poisson's ratio (0.3), *E* is the E-field, *d*_33_ and *d*_31_ is the piezoelectric coefficients, and *λ* is the saturation magnetostriction coefficient (34 ppm for L1_0_-FePt films).^28,29^ The *d*_33_ and *d*_31_ under different temperatures are given by Peng and Li,^13,24^ and we set *E* = 10 kV cm^−1^, and then the strain induced effective field *H*_s_ was calculated to be 999.47 Oe at 350 K, which is higher than *H*_s_ of 809.28 Oe at 275 K. Because of the large strain induced effective field *H*_s_ and weak PMA of L1_0_-FePt films at 350 K,^19^ coercivity *H*_c_ has a larger change, which reveals E-field has an improved regulating effect on PMA of PMN–PT/FePt heterostructures at higher temperature.

**Fig. 4:**
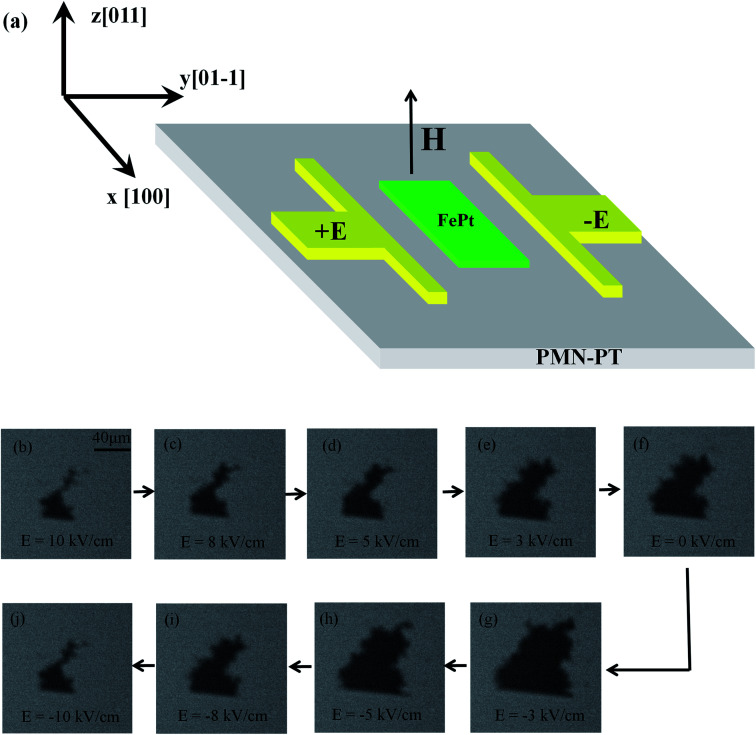
(a) Schematic of the MOKE measurement. (b–j) present MOKE images of E-field controlled magnetization switching of the PMN–PT/FePt heterostructure. The white and black areas of MOKE images stand for the magnetization up and down, respectively.

For verifying the E-field controlled PMA of PMN–PT/FePt heterostructures, we observe the magnetization switching of L1_0_-FePt films under different E-fields directly by the MOKE microscope. Due to the restriction of the MOKE microscope, we can only observe the magnetization switching on rectangular L1_0_-FePt films instead of Hall bar at 300 K. The MOKE measurement is schematically shown in Fig. 4(a). During the measurement, a fixed magnetic field of 367 Oe with perpendicular direction is applied, where the magnetization begins to switch. Compared with other magnetic fields (Fig. S3, ESI†), the magnetic field of 367 Oe enables us to observe the electric field-controlled magnetization switching clearly. Fig. 4(b)–(j) present the MOKE images of E-field-controlled magnetization switching of L1_0_-FePt films. The white and black areas of MOKE images stand for the magnetization up and down, respectively. With the E-field scanning from 10 kV cm^−1^ to −10 kV cm^−1^, the small black area first apparently expands to maximum and then reduces to the original state, the magnetization direction of which is opposite to external magnetic field. According to the discussion above, the L1_0_-FePt films suffer the tensile strain at *E* = ±10 kV cm^−1^ along the [011] direction, which reduces the PMA of L1_0_-FePt films and makes the magnetization switch easily under the fixed magnetic field. Therefore, the area of magnetization down is small. The L1_0_-FePt films suffer the compressive strain along the [011] direction at *E* = −3 kV cm^−1^. Compared with tensile strain, compressive strain exerts opposite effects on L1_0_-FePt films, which enhances the PMA and expands the area of magnetization down. The magnetization switching observed by the MOKE microscope under different E-fields gives us a compelling evidence of the E-field-controlled PMA of PMN–PT/FePt heterostructures.

**Fig. 5:**
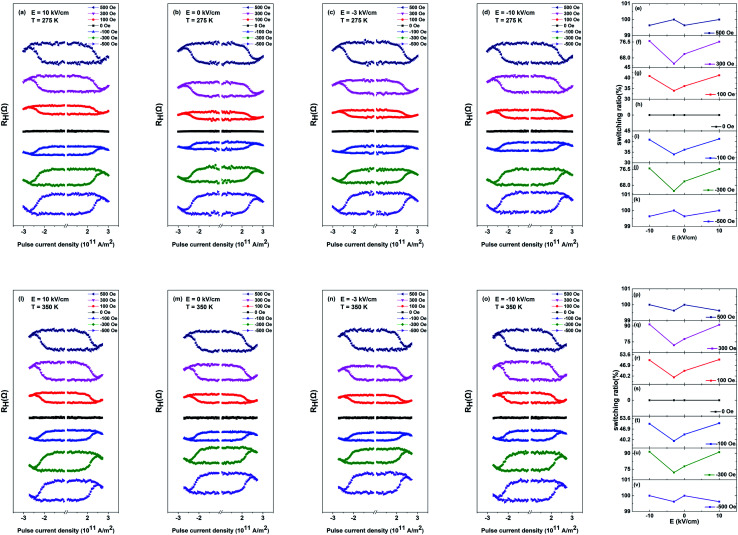
(a–d) *R*_H_–*I* loops of L1_0_-FePt films with different *H*_x_ under varying E-field (*E* = 10, 0, −3, −10 kV cm^−1^) at 275 K, respectively. (e–k) The dependence of the magnetization switching ratio on the applied E-field with different *H*_x_ (500 Oe, 300 Oe, 100 Oe, 0 Oe, −100 Oe, −300 Oe, −500 Oe) at 275 K, respectively. (l–o) *R*_H_–*I* loops of L1_0_-FePt films with different *H*_x_ under varying E-field (*E* = 10, 0, −3, −10 kV cm^−1^) at 350 K, respectively. (p–v) The dependence of magnetization switching ratio on the applied E-field with different *H*_x_ (500 Oe, 300 Oe, 100 Oe, 0 Oe, −100 Oe, −300 Oe, −500 Oe) at 350 K, respectively.

**Fig. 6:**
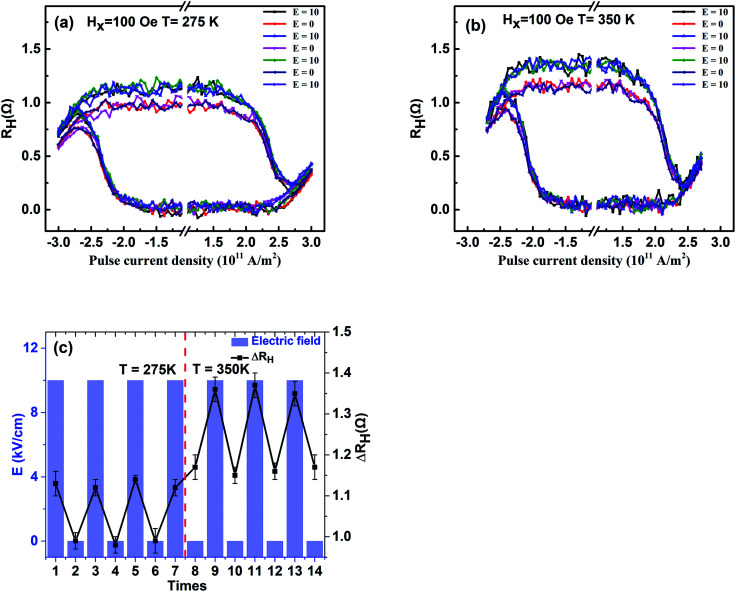
(a) and (b) show the *R*_H_–*I* curves under alternate E-field with *H*_x_ of 100 Oe at 275 K and 350 K, respectively. The unit of E is kV cm^−1^. (c) Dependence of Δ*R*_H_ on alternate E-field at 275 K and 350 K.

According to the discussion above, PMA of PMN–PT/FePt heterostructures can be manipulated more effectively by E-field at high temperatures. It is rational to explore temperature assistance of E-field-controlled SOT-based current induced magnetization switching of PMN–PT/FePt heterostructures. Fig. 5(a)–(d) show the *R*_H_–*I* loops of L1_0_-FePt films with different *H*_x_ under varying E-field at 275 K. The *R*_H_–*I* loops with *H*_x_ of ±100 Oe and ±300 Oe show partial magnetization switching and *R*_H_–*I* loops with *H*_x_ of ±500 Oe show fully magnetization switching. When *H*_x_ is ±100 Oe or ±300 Oe, *H*_x_ < *H*_DMI_, the external magnetic field *H*_x_ is not sufficient to overcome the DMI effective field *H*_DMI_. Therefore, the current-induced magnetization cannot be switched totally. Fig. 5(e)–(k) summarize the dependence of the magnetization switching ratio on applied E-field with different *H*_x_ at 275 K. With the E-field scanning from 10 kV cm^−1^ to −10 kV cm^−1^, the magnetization switching ratio first decreases from 41.8% to 33.9% and then rises back to 41.2% and has a change of 21.5% with *H*_x_ of ±100 Oe at 275 K, and magnetization switching ratio with *H*_x_ of ±300 Oe at 275 K shows the same trend and has a change of 18.6%. However, magnetization switching ratio with *H*_x_ of ±500 Oe at 275 K does not change under different E-fields, which indicates the effects of strain on magnetization can be ignored with *H*_x_ of ±500 Oe. The compressive strain along the [011] direction at *E* = −3 kV cm^−1^ enhances PMA, which results in difficult magnetization switching and a small magnetization switching ratio. The tensile strain at *E* = ±10 kV cm^−1^ results in a large magnetization switching ratio. Fig. 5(l)–(o) show the *R*_H_–*I* loops of L1_0_-FePt films with different *H*_x_ under varying E-field at 350 K. Fig. 5(p)–(v) summarizes the dependence of the magnetization switching ratio on applied E-field with different *H*_x_ at 350 K. Compared with change in the magnetization switching ratio induced by E-field at 275 K, the magnetization switching ratio has an improved change of 28.2% with *H*_x_ of ±100 Oe and an improved change of 27.2% with *H*_x_ of ±300 Oe at 350 K under applied E-field, which is attributed to the large strain-induced effective field *H*_s_ and weak PMA of L1_0_-FePt films at 350 K. To check the stability of the Hall bar, Fig. 6(a) and (b) show the *R*_H_–*I* curves under alternate E-field with *H*_x_ of 100 Oe at 275 K and 300 K, respectively. Fig. 6(c) summarizes the dependence of Δ*R*_H_ on alternate E-field with *H*_x_ of 100 Oe at 275 K and 300 K. The change of Δ*R*_H_ is stable under alternate E-field at 275 K and 300 K, which suggests that the E-field modulated SOT-based current-induced magnetization switching of PMN–PT/FePt heterostructures is more effective and steady with small *H*_x_ at a high temperature.

**Fig. 7:**
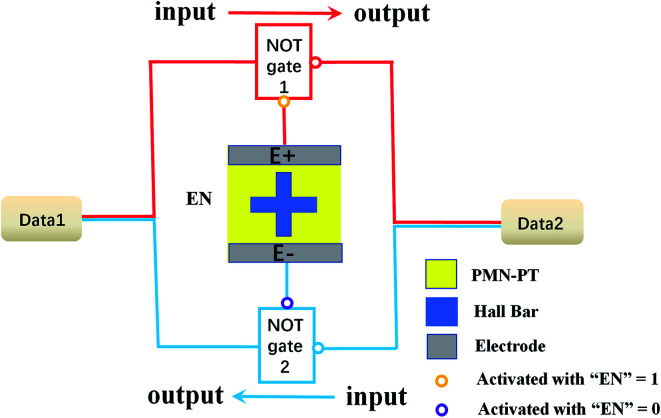
Schematic designs of an SOT-based logical circuit composed by two NOT gates and an output control “EN”, where the Hall bar is output control EN.

An E-field controlled SOT-based logical circuit consists of two NOT gates and an output control “EN” is designed, which realize the bidirectional transmission of data, as shown in Fig. 7. Both Data1 and Data2 can be used as inputs or outputs. The Hall bar is regarded as the “EN”, which is a switch for NOT gates. When “EN” = 1, the NOT gate 1 is activated and outputs of red circuit are consistent with the logic of NOT gate 1, while the NOT gate 2 is deactivated. Continuously, when “EN” = 0, the NOT gate 2 is activated and outputs of blue circuit are consistent with the logic relationship of NOT gate 2, and the NOT gate 1 is deactivated. According to our studies mentioned above, when the *H*_x_ is 100 Oe and E-field is “on” (10 kV cm^−1^), Δ*R*_H_ of the Hall bar is 1.13 Ω, which stands for the high electrical level and “EN” = 1. With “EN” = 1, the NOT gate 1 is activated. The data is transmitted from left to right and we can get Data2 = −Data1. On the contrary, when the E-field is “off” (0 kV cm^−1^), Δ*R*_H_ of Hall bar is 0.99 Ω, which stands for the low electrical level and “EN” = 0. With “EN” = 0, the NOT gate 2 is activated. The data is transmitted from right to left and we can get Data1 = −Data2. Consequently, we summarize the outputs of the logical circuit, which depend on the inputs and “EN”, as shown in Table 1. We realize the bidirectional transmission of data with the help of the PMN–PT/FePt heterostructure. An E-field controlled SOT-based logical circuit can be widely used in computer, digital control, communication, automation and instrument.

**Table 1:** Truth table of an SOT-based logical circuit based on the *R*_H_–*I* loops shown in Fig. 6 and the design of Fig. 7, where D1 is Data1 and D2 is Data2. Data goes from input to output

EN = 1			EN = 0		
Input D1	1	0	Input D2	1	0
Output D2	0	1	Output D1	0	1

## Conclusions

In conclusion, L1_0_-FePt films were deposited on the PMN–PT(011) substrate. While the temperature increases, the coercivity *H*_c_ and critical current density *J*_c_ decrease monotonically because of the large strain-induced effective field *H*_s_ and weak PMA of L1_0_-FePt films at 350 K, E-field has an improved regulating effect on PMA- and SOT-based current-induced magnetization switching of PMN–PT/FePt heterostructures. Magnetization switching observed by an MOKE microscope confirms that PMA can be controlled by E-field. In addition, change in the anomalous Hall resistance Δ*R*_H_ is modulated reversibly by alternate E-field under different temperatures, which enables us realize an E-field-controlled SOT-based logical circuit and the bidirectional transmission of data. These new discoveries offer us an avenue to manufacture potential spintronic devices and technologies by the E-field modulation of PMN–PT/FePt heterostructures at different temperatures.

## Author contributions

These authors contributed equally to this work.

## Conflicts of interest

There are no conflicts to declare.

## Supplementary Material

RA-011-D1RA00919B-s001
