# Supplementary material for: Temperature assistance of electric field-controlled spin–orbit torque-based magnetization switching in PMN–PT/FePt heterostructures
Source: RSC Adv. 2021 Mar 24;11(20):12043–50. doi: 10.1039/d1ra00919b (PMC8697033; doi:10.1039/d1ra00919b)
Supplement: RA-011-D1RA00919B-s001 [file RA-011-D1RA00919B-s001.pdf]

Electronic Supplementary Material (ESI) for RSC Advances.
This journal is © The Royal Society of Chemistry 2021

Electronic Supplementary Information

Temperature assistance of electric field controlled spin orbit torque based magnetization switching in PMN-PT/FePt heterostructures

Qi Guo[a], Zhicheng Wang[a*]

[a] School of Materials Science and Engineering, Taiyuan University of Science and Technology, Taiyuan 030024, China

E-mail: zcwang@tyust.edu.cn

The roughness of PMN-PT (011) substrate has a very important impact on the strain transfer efficiency from PMN-PT (011) substrate to FePt films. So we check the root mean square roughness value of PMN-PT (011) substrate, which is 0.51 nm as shown in Figure S1 by AFM. The roughness of PMN-PT(011) substrate is comparable with that of the thermally oxidized Si substrate, which is about 0.60 nm.

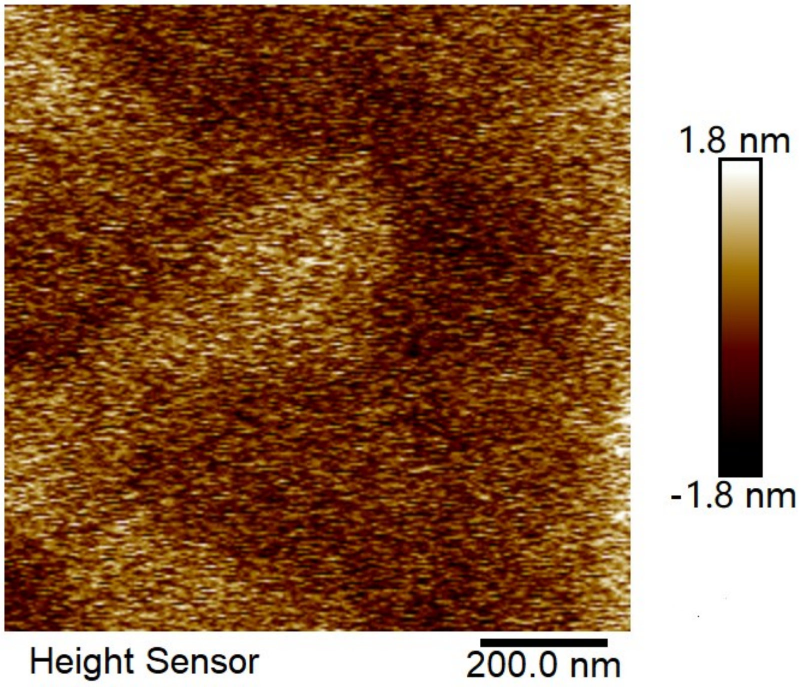

Figure S1. AFM image of the PMN-PT (011) substrate.

Figure S2 shows the enlarged R_H–H loops of Figure 2 (a). Change of anomalous Hall resistance ΔR_H demonstrates a slight reduction from 2.74 Ω to 2.67 Ω with temperature increasing. According to Chen,[1] ΔR_H characterizes the out of plane magnetization of $L1_0$-FePt films. Due to the increasing of thermal fluctuation and decreasing of magnetic anisotropy energy with temperature increasing, the out of plane magnetization is expected to decrease with temperature increasing so that the corresponding ΔR_H decreases. However, the temperature changes in a small range from 275K to 350K, which results in a slight reduction of ΔR_H. Our results are consist with the experimental results reported by Chen and Lim.[2,3]

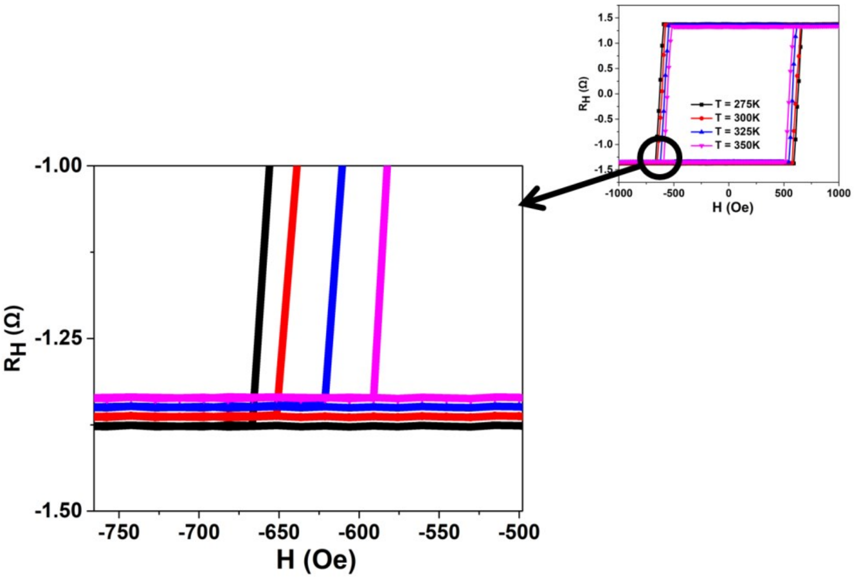

Figure S2. The enlarged R_H–H loops of Figure 2 (a).

For verifying the electric field controlled PMA of PMN-PT/FePt heterostructures, we observe the magnetization switching of $L1_0$-FePt films by MOKE microscope under electric field, the magnetization of $L1_0$-FePt films aligns positive or negative perpendicular direction. So we need a perpendicular magnetic field to observe the magnetization switching.

Before the MOKE measurement, the $L1_0$-FePt films were magnetized under a large negative perpendicular magnetic field (-6000 Oe) to ensure the magnetization of $L1_0$-FePt films orientated in negative direction. Then, we increase the magnetic field until magnetization switching appears. When the magnetic field increased to 367 Oe, the magnetization switching can be observed in MOKE microscope. Part of magnetization orientated in negative direction and others orientated in positive direction under magnetic field of 367 Oe, which enable us to observe the electric field controlled magnetization switching clearly. If the magnetic field is smaller than 367 Oe, the magnetization orientates in negative direction and does not begin to switch shown in Fig. S3(a) and (b). If the magnetic field is larger than 367 Oe, the magnetization switches in positive direction totally shown in Fig. S3 (c) and (d). In the both situations, magnetic field dominates, which shield the effect of electric field, and we can not observe the electric field controlled magnetization switching. So, we can only observe the magnetization switching under electric field with the applied fixed magnetic field of 367 Oe in perpendicular direction.

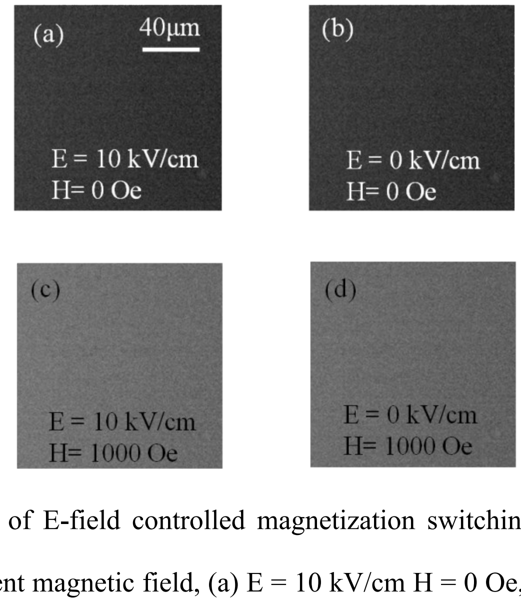

Fig.S3 MOKE images of E-field controlled magnetization switching of the PMN-PT/FePt heterostructure under different magnetic field, (a) E = 10 kV/cm H = 0 Oe, (b) E = 0 kV/cm H = 0 Oe, (c) E = 10 kV/cm H = 1000 Oe, (d) E = 0 kV/cm H = 1000 Oe,. The white and black areas of MOKE images stand for the magnetization up and down, respectively.

References for Electronic Supplementary Information

1 M. Chen, Z. Shi, W. J. Xu, X. X. Zhang, J. Du and S. M. Zhou, *Appl. Phys. Lett.*, 2011, **98**, 082503.

2 S. Chen, D. Li, B. Cui, L. Xi, M. Si, D. Yang and D. Xue, *J. Phys. D. Appl. Phys.*, 2018, **51**, 095001.

3 G. J. Lim, W. L. Gan, W. C. Law, C. Murapaka and W. S. Lew, *J. Magn. Magn. Mater.*, 2020, **514**, 167201
